# Chronic heat stress part 1: Decrease in egg quality, increase in cortisol levels in egg albumen, and reduction in fertility of breeder pekin ducks

**DOI:** 10.3389/fphys.2022.1019741

**Published:** 2022-11-11

**Authors:** E. M. Oluwagbenga, V. Tetel, J. Schober, G. S. Fraley

**Affiliations:** Animal Sciences, Purdue University, West Lafayette, IN, United States

**Keywords:** climate change, heat stress, welfare, cortisol, corticosterone, egg quality, pekin duck

## Abstract

Global warming poses detrimental effects on poultry production leading to substantial economic losses. The goal of our experiment was to test the hypothesis that heat stress (HS) would alter welfare and egg quality (EQ) of breeder ducks. Furthermore, we wanted to test if HS would increase cortisol levels in egg albumen. Adult Pekin ducks were randomly assigned to two different rooms at 85% lay with 60 hens and 20 drakes per room. Baseline data including body weight, body condition scores (BCS), and egg production/quality were collected the week preceding heat treatment. Ducks were subjected to cyclic HS of 35°C for 10h/day and 29.5°C for the remaining 14h/day for 3 weeks while the control room was maintained at 22°C. Eggs were collected daily and analyzed weekly for quality assessment, and for albumen glucocorticoid (GCs) levels using mass spectrometry. One week before the exposure to HS, 10 hens and 5 drakes were euthanized and the same number again after 3 weeks and birds necropsied. Data analyses were done by 1- or 2-way ANOVA as appropriate with a Tukey-Kramer *post hoc* test. BCS were analyzed using a chi-squared test. A *p* ≤ 0.05 was considered significant. Circulating levels of corticosterone were significantly (*p* < 0.01) elevated at week 1 only in the HS hens. The circulating levels of cortisol increased significantly at week 1 and 2 (*p* < 0.05), and week 3 (*p* < 0.01) in the hens and at weeks 2 and 3 only (*p* < 0.05) in the drakes. Feather quality scores (*p* < 0.01), feather cleanliness scores (*p* < 0.001) and footpad quality scores (*p* < 0.05) increased significantly in the HS group. HS elicited a significant (*p* < 0.001) decrease in egg production at weeks 1 and 3. Hens in the HS group showed significantly decreased BW (*p* < 0.001) and number of follicles (*p* < 0.05). Shell weight decreased significantly at week 1 only (*p* < 0.05) compared to controls. Yolk weight decreased significantly at week 3 (*p* < 0.01) compared to controls. HS elicited a significant increase in albumen cortisol levels at week 1 (*p* < 0.05) and week 3 (*p* < 0.05). Thus, cortisol may provide critical information to further understand and to improve welfare.

## Introduction

Environmental stressors are major concerns facing poultry production. HS is one of the most prominent, having detrimental effects on the production, performance, welfare, meat quality, immunity, and egg quality of poultry (Ebeid et al., 2012; Honda et al., 2015; Barrett et al., 2019; He et al., 2019). According to St-Pierre et al. (2003), the United States incurred an annual loss of $1.69–2.36 billion in the whole livestock industry of which $128–165 million occurs in poultry industry because of HS. Poultry is known as an excellent source of protein in the form of meat and egg (Nawab et al., 2018) and poultry meat accounts for 34.3% of global meat production in 2012 (Pawar et al., 2016). Generally, poultry are more susceptible to HS because of their high body temperature and lack of sweat glands for heat dissipation (Wolfenson et al., 2001). A growing number of studies have exposed meat type and laying ducks to high temperature to assess the effect on performance and reproductive parameters. The effects of heat stress on ducks include decreased daily feed intake and decreased egg quality in shelducks. Studies have suggested that heat stress reduces fertility due to decreased oviduct length and number of ovarian follicles in shelducks (Ma et al., 2014), however actual fertility data is lacking. Heat stress also elicits production and biochemical effects in ducks such as decreased growth performance and breast meat quality in pekin ducks (Sun et al., 2019), increased mRNA expression of heat shock protein and inflammatory factors in muscovy and pekin ducks (Zeng et al., 2014), and decrease in hypothalamic expression of antioxidant and pro-oxidant enzymes genes in laying shanma ducks (Luo et al., 2018). Heat stress has also been suggested to increase the stress response as evidenced by an increase in adrenal gland weight in pekin ducks (Hester et al., 1981), HS activates the sympathetic adrenomedullary (SAM) and hypothalamic-pituitary-adrenal (HPA) axes that lead to the secretion of catecholamines and glucocorticoids (GC) respectively (Nagarajan et al., 2017). Several studies have shown that acute, chronic, and cyclic HS all affect circulating levels of GC (reviewed by Scanes, 2016). Increased circulating levels of GC induces gluconeogenesis, cell trafficking and proliferation, cytokine secretion, and antibody production that can affect immunity, promote inflammation and lead to hypersensitive HPA axis (Shini et al., 2008; Lara and Rostagno, 2013). In addition, HS can dysregulate the hypothalamic-pituitary-gonadal (HPG) axis thereby altering the secretion of gonadotropin releasing hormone (GnRH) from the hypothalamus, and luteinizing hormone (LH) and follicle stimulating hormone (FSH) from the anterior pituitary gland (Lu et al., 2021). HS also depresses the gonads thereby reducing the secretion of testosterone from testes and estradiol from the ovaries, causing a decline in ova maturation, sperm quality, morphology, mobility, and egg penetration (reviewed by Ayo et al., 2011). Thereby the effect of HS on the HPG axis can lead to a decrease in fertility.

The purpose of our study was to evaluate the effects of HS on egg quality, the stress response, fertility, and welfare of pekin ducks. To achieve this, we treated 80 adult Pekin ducks at 85% lay to HS and an equal number as controls. Our results suggest that HS elicited a selective deposition of cortisol, not corticosterone, into the egg albumen. Further, HS decreased shell and yolk weights, caused a decline in duck welfare, and affected ovarian but not testes morphometrics.

## Materials and methods

### Animals

160 developer pekin ducks of approximately 20 weeks of age were obtained from Maple Leaf Farms Inc. (Leesburg, IN) and randomly allocated into 2 rooms in equal numbers at industry standard density. They were allowed *ad lib* access to water and feed for 8 h per day as per standards (Chen et al., 2021). We utilized 60 hens and 20 drakes per treatment. None of the ducks had any prior exposure to HS. The ducks were placed in the single rooms with an 18:6 light cycle, temperature of 20–22°C for both treatment groups until 85% lay (∼35 weeks of age). Water nipple lines (5 ducks per nipple) were placed over a pit covered with raised plastic flooring, and the remaining area of the rooms were covered with pine shavings and added to or replaced as necessary at the same time for both rooms. Nest boxes were placed along one wall of the room with 4 hens per nest box as per industry standards (Pfaus et al., 2001; Chen et al., 2021). All procedures were approved by the Purdue Animal Care and Use Committee (PACUC # 2109002195).

### Experimental design

At 85% lay, the 2 rooms were randomly allocated as control or HS group such that there is one room per treatment group. The HS group was subjected to cyclic temperature of 35°C for 10h/day and returned to 29.5°C for the remaining 14h/day for 3 weeks while the control room was modified to an industry-standard temperature of 22°C. Both rooms had data loggers (Hobo, Onset Inc.) for temperature and humidity that were placed to monitor both variables at the level of the ducks’ heads. Ammonia readings were taken twice per week using a NH_3_ meter (Forensic Detectors, NH3000).

### Sample collection and preparation

Blood smears were collected for HLR on weeks 0, 1, 2, and 3 relatives to the onset of heat treatment from different ducks at each collection (n = 6/sex/treatment/week), with the week preceding heat treatment designated as 0 and analyzed by a certified pathologist in a diagnostics laboratory unaware of the treatment groups. Body condition scores (BCS) (Fraley et al., 2013; Karcher et al., 2013; Colton et al., 2014) such as foot pad quality scores, eyes scores, nostrils scores, feather cleanliness and quality scores were also assessed on weeks 0, 1, 2 and 3 for all ducks at each assessment (n ∼ 65/treatment) using the scoring rubric shown in Table 1.

**TABLE 1:** Body condition scoring rubric for welfare assessment^a^.

Structure	Score level	Description
Eyes	0	**Best:** Eyes clear, clean, and bright.
	1	**Moderate:** Dirt and/or staining around the eye area. Any evidence of wet eye ring or inflamed eye lid.
	2	**Worst:** Eyes sealed shut with or without conjunctivitis.
Nostrils	0	**Best:** Nostrils with clean and clear air passageways.
	1	**Dirty:** Nostril air passageways block with dust or mucus.
Feather Cleanliness	0	**Best:** Clean and unstained breast, back feathers or down depending on age.
	1	**Dirty:** Adhering manure or staining on down or feathers.
Feather Quality	0	**Best:** good feather coverage for age—down in younger and feathers for developing and older birds
	1	**Moderate:** Some evidence of feather picking, down and/or feather damage less than 2 cm^2^.
	2	**Worst:** Feathers/down damaged, short, and stubbly. Large patchy feathers/down over back greater than 2 cm^2^ and/or evidence of severe feather picking (presence of blood on back, tail, neck, or wings).
Foot pad	0	**Best:** Heel and toe pads free of any lesions or ingrained dirt.
	1	**Moderate:** Dirt pervades the heel or toe pads and skin papillae raised typically dark brown on heel or toe pads. Lesions covering less than 50% of heel or toe pad. Free of any bloody lesions.
	2	**Worst:** lesions or callouses cover 50% or more of heel or toe pad, any bleeding lesions.

^a^ Adapted from Fraley et al. (2013) and Karcher et al. (2013).

Blood samples were collected from the ducks’ tibial veins at the same time as blood smears on weeks 0, 1, 2, and 3 relative to the onset of HS from randomly selected ducks at each time point (n = 6/sex/treatment/week) and placed into a serum separator tubes, centrifuged, and the serum was stored at −20°C until assayed by ELISA for GC (Tetel et al., 2022a; Tetel et al., 2022b). Daily eggs laid (*n* = 35–60) were counted and 3 consecutive day eggs (thus *n* = 3 per time group) were averaged to reduce daily lay variability and compared between treatment groups beginning 1 week prior to HS treatment (*n* = 3/group/treatment): groups -1 and 0 (week preceding heat stress), groups 1 and 2 (week 1), groups 3 and 4 (week 2), and groups 5, 6 and 7 (week 3). All eggs from each treatment group were also collected daily over the 4-week period and assessed for egg quality weekly (*N* = 33–44 per daily collection per week) as described below. Yolk and albumen (*N* = 10 each per treatment group) samples were collected into tubes during egg quality assessment and stored at -20°C until assayed for GC using mass spectrometry. Ducks were weighed on days 0 and 21 relative to the onset of HS. One week before the exposure to HS, 10 hens and 5 drakes were euthanized and the same number again after 3 weeks of HS or control exposures using pentobarbital (Fatal Plus, 396 mg/ml/kg) and birds necropsied. Spleen, testes, and liver were collected and weighed, the number of maturing follicles recorded, and a final blood sample was obtained and treated as described above.

### Egg incubation

Eggs from the last 3 days of the treatment were collected from both groups (*n* = 127 for control and *n* = 113 for HS group). They were stored at 4°C for 5 days. After storage, the eggs were sorted and incubated per industry standard (Chen et al., 2021). Candling was done on day 10 and the number of fertile eggs was counted and calculated as percentage for fertility. Eggs hatched on days 28 and 29 and number of dead embryos were counted and expressed as a percentage of embryo mortality. Number of hatched chicks were counted and calculated for hatchability.

### ELISA for glucocorticoids

The kits utilized for this project were from Cayman Chemicals (corticosterone: kit #16063; cortisol kit #560360) and the assays were run according to the manufacturer’s recommendations. Details of the kit verification have been reported previously (Tetel et al., 2022a; Tetel et al., 2022b). Plates were incubated with samples overnight at 4°C. Plates were developed for 90 min and were read at 405 nm (SynergyLx, Biotek).

### Egg quality assessments

Eggs were identified by room and collected on weekly basis for the duration of the trial for quality assessment, refrigerated at 4°C overnight, and weighed before analyses (*n* = 33–44/treatment/week). Shell compression strength was analyzed using the TA.XT Plus Texture Analyzer (Texture Technologies, Hamilton, MA) with a 10 kg load cell. The egg was placed on its side using the egg holder (TA-650) and compressed with the compression disc (TA-30). The albumen height was measured with a micrometer and the Haugh unit was calculated. The yolk was separated and weighed, then the vitelline membrane compression strength was analyzed using TA.XT Plus Texture Analyzer with a 500 g load cell. The yolk was placed in a petri dish and compressed with the compression disc. Albumen and yolk samples were collected into tubes and stored at—20°C for GC assays. The eggshells with intact membranes were washed, placed on trays, and dried at room temperature for 48 h. When the eggshells were fully dried, the weights were obtained, and the shell thickness was measured at three different points around its equator and the values averaged. Data from 1 week before treatment were classified as 0 and from weeks 1, 2 and 3 after the exposure to HS.

### Mass spectrometry for yolk and albumen glucocorticoids

Yolk (*n* = 10/treatment/week) and albumen (n = 10/treatment/week) samples from the same egg were stored at −20°C before extraction and analysis. At the time of analysis, each albumen sample was thawed, 500 mg of albumen was transferred to an extraction tube while 5 g of each thawed yolk sample was transferred to an extraction tube. The samples were extracted according to a previous report with minor modifications (Caulfield and Padula, 2020). To each sample, 10 µL of an internal standard mixture containing 5 ng of deuterated corticosterone (d8-corticosterone solution in methanol) and 1 ng of deuterated cortisol (d4-cortisol solution in methanol) was added to the albumen and yolk samples and vortexed for 1 minute. The internal standards d8-corticosterone (# C695702) and d4-cortisol (# C696302) were purchased from Toronto Research Chemicals (Ontario, Canada). Next, 2.5 ml and 5 ml of acetonitrile +1% formic acid was added to albumen and yolk respectively to extract corticosterone and cortisol. The samples were vortexed for 10 min and centrifuged at 3,220 g for 5 min at room temperature. The supernatants were collected and transferred to new tubes. The extract was then washed with equal volumes of hexane. The hexane was discarded, and the bottom phase was collected and dried using nitrogen gas. The pellet was then reconstituted in 0.1 ml of methanol, followed by 0.9 ml of water. The samples were mixed well by vortexing before loading onto a Water’s HLB PRIME solid-phase extraction cartridge, 1cc, 30 mg (part number 186008055; Waters Corp., Milford MA). The cartridges were washed with 1 ml of water followed by 1 ml of hexane. The samples were eluted with 2 volumes of 1 ml of ethyl acetate. This fraction was dried using a nitrogen stream or speed vacuum. The samples were then stored at -80°C until ready for analysis.

For analysis, each sample was derivatized with 50 µL of Amplifex keto reagent (# 4465962, AB Sciex, Framingham, MA) according to the kit directions just before instrument analysis. After 1 hour 10 µl of water was added and the sample mixed well before transferring to an autosampler vial for analysis by LC/MS/MS. An Agilent 1260 Rapid Resolution liquid chromatography (LC) system coupled to an Agilent 6470 series QQQ mass spectrometer (MS/MS) was used to analyze corticosterone and cortisol in each sample (Agilent Technologies, Santa Clara, CA). An Agilent Eclipse plus C18 2.1 mm × 50 mm, 1.8 µm column was used for LC separation. The buffers were (A) water +0.1% formic acid and (B) acetonitrile +0.1% formic acid. The linear LC gradient was as follows: time 0 min, 10% B; time 1.0 min, 10% B; time 1.5 min, 25% B; time 21.5 min, 35% B; time 22 min, 100% B; time 23 min, 100% B; time 24 min, 10% B; time 30 min, 10% B. The flow rate was 0.3 ml/min. Corticosterone eluted at 6.6 min and cortisol at 5.8 min. Multiple reaction monitoring was used for MS analysis. The data were acquired in positive electrospray ionization (ESI) mode (Tetel et al., 2022a; Tetel et al., 2022b). The jet stream ESI interface had a gas temperature of 325°C, gas flow rate of 8 L/min, nebulizer pressure of 45 psi, sheath gas temperature of 250°C, sheath gas flow rate of 7 L/min, a capillary voltage of 4000 V in positive mode, and nozzle voltage of 1000 V. The ΔEMV voltage was 500 V. Agilent Masshunter Quantitative analysis software was used for data analysis (version 10.1). For quantitation of corticosterone/d8-corticosterone, the transition 461.3 → 402.2/469.3 → 410.2 was used. For cortisol/d4-cortisol, the transition 477.3 → 418.3/481.3 → 422.3 was used.

### Statistical analyses

The duck was considered the statistical unit. Given the limitations of our model design, we ran power analyses following a simulation approach (i.e., simulating data with parameters taken from previous studies) to establish the sample size per each treatment variable. All our power analyses were run in MacJMP SAS (JMP Pro 15). We ran four power analyses considering four indicators of our response variables: BCS, blood cell counts, hormone levels, and egg quality. We found that the sample sizes described above would provide us 85% power to detect significant effects (*p* < 0.05) taking into account inter-duck variability. All data were analyzed by 1-, or 2 -way ANOVA as appropriate. Body condition scores were analyzed using a chi-squared test. *Post hoc* analyses were done by Tukey Kramer pairwise comparison test. A *p* < 0.05 was considered significant.

## Results

### Environmental conditions

Data loggers confirmed cyclic heat temperatures in the HS room and control conditions. Bedding was replaced as needed in the HS room, and in control room at the same time. Regardless, analyses of NH_3_ showed that control room had below measurable levels, while the HS room averaged 12.5+/− 2.36 ppm during the 3 weeks of HS. The necessary use of electric heating units on our farm facility led to low relative humidity levels with the control room averaging 37.4 +/− 2.43% and the HS room averaging 64.7 +/− 4.66%. The differences in NH_3_ and humidity levels in between groups are possible confounding factors.

### Serum glucocorticoids

**FIGURE 1:**
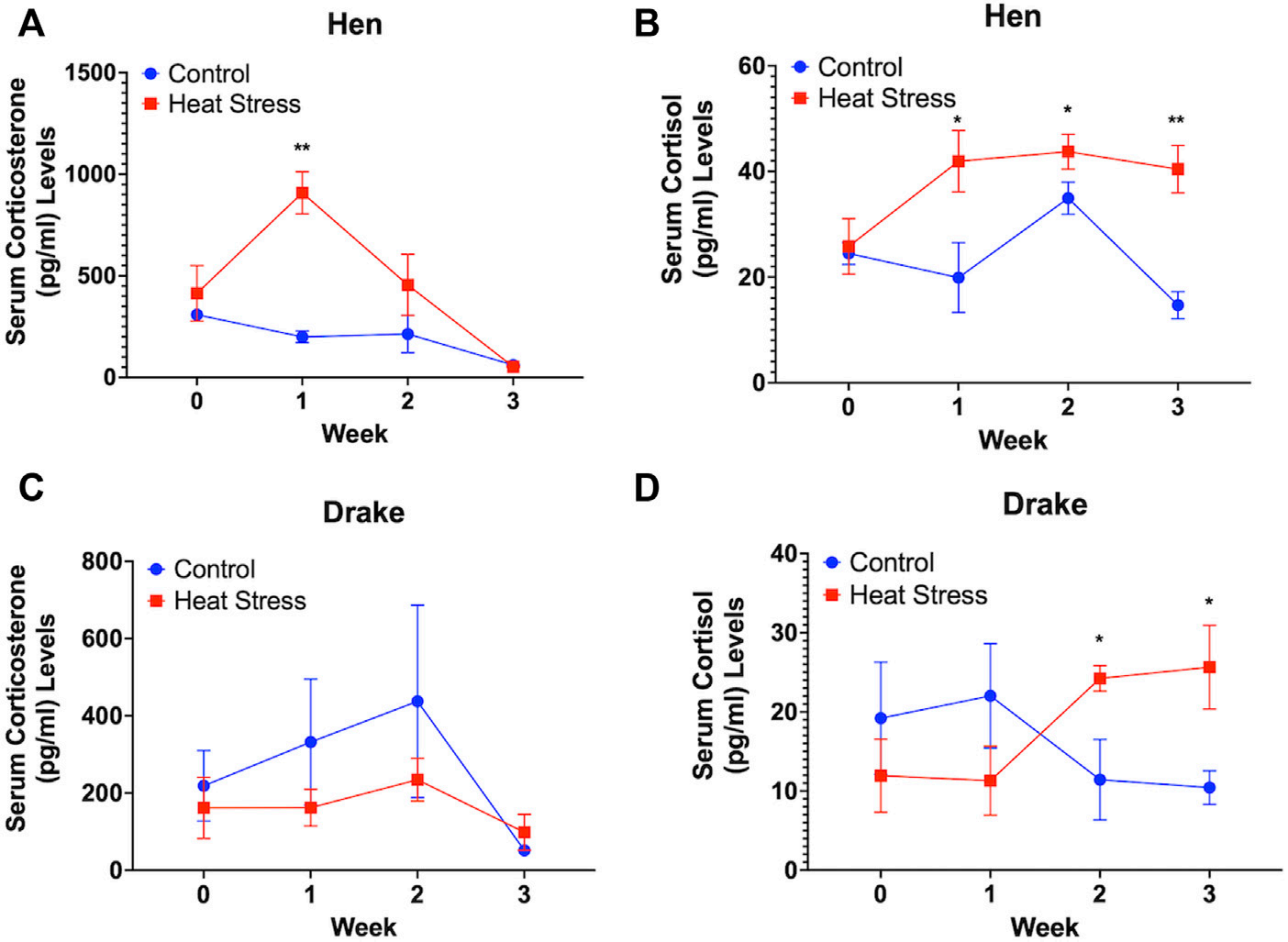
Serum corticosterone **(A)** and cortisol levels **(B)** in Hens and serum corticosterone **(C)** and cortisol **(D)** in Drakes exposed to cyclic heat stress (35°C for 10h/day and 29.5°C for the remaining 14h/day) or control. Data shown are means ± SEM, *n* = 6/sex/treatment/week. Ducks exposed to heat stress showed a significant increase in serum cortisol levels compared to controls at weeks 1, 2, and 3 for hens and weeks 2 and 3 for drakes after the onset of treatment. Only hens exposed to heat stress showed a significant increase in serum corticosterone levels compared to controls at week 1 only after onset of treatment. * = *p* < 0.05, ** = *p* < 0.01.

Circulating levels of corticosterone in the hens were significantly (*p* < 0.01) elevated at week 1 only in the HS group (Figure 1A) while the circulating levels of cortisol increased significantly at week 1 (*p* < 0.05), week 2 (*p* < 0.05), and week 3 (*p* < 0.01) in the hens compared to the controls for each hormone (Figure 1B). In contrast, there was no significant increase in the circulating levels of corticosterone in drakes in either group (Figure 1C), while HS significantly increased the serum levels of cortisol at week 2 and 3 only (*p* < 0.05) in the drakes compared to control drakes (Figure 1D).

### Welfare assessment

**FIGURE 2:**
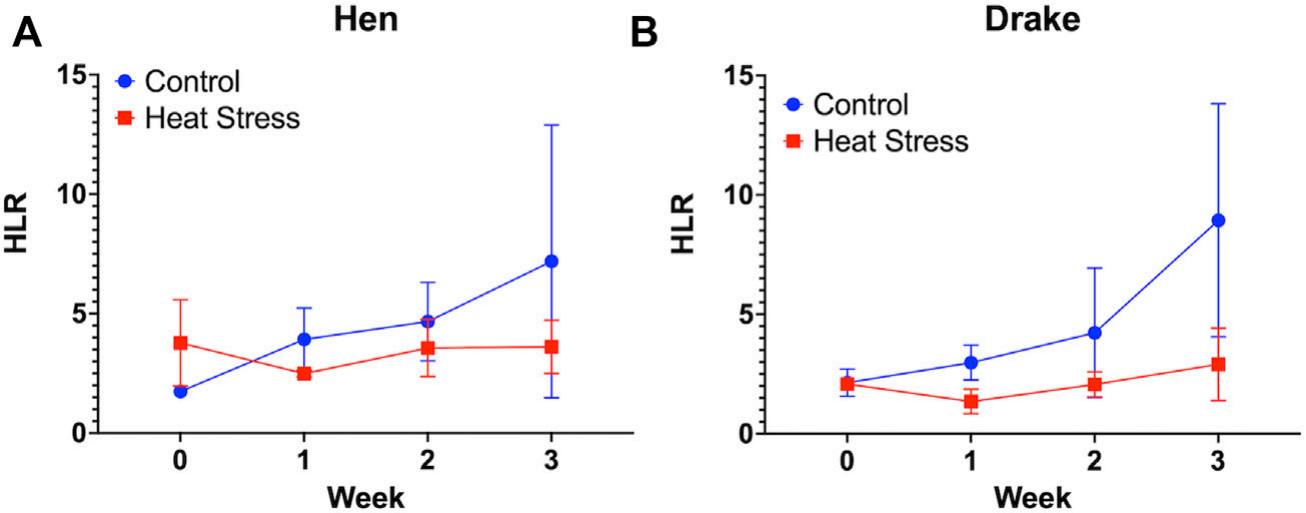
Heterophil and Lymphocyte ratio in Hens **(A)** and Drakes **(B)** exposed to cyclic heat stress (35°C for 10h/day and 29.5°C for the remaining 14h/day) or control. Data shown are means ± SEM, *n* = 6/sex/treatment/week. No significant difference were observed in the HLR of both drake and hen, however elevated levels in both groups suggest a hypersensitivity to the handling procedures.

**FIGURE 3:**
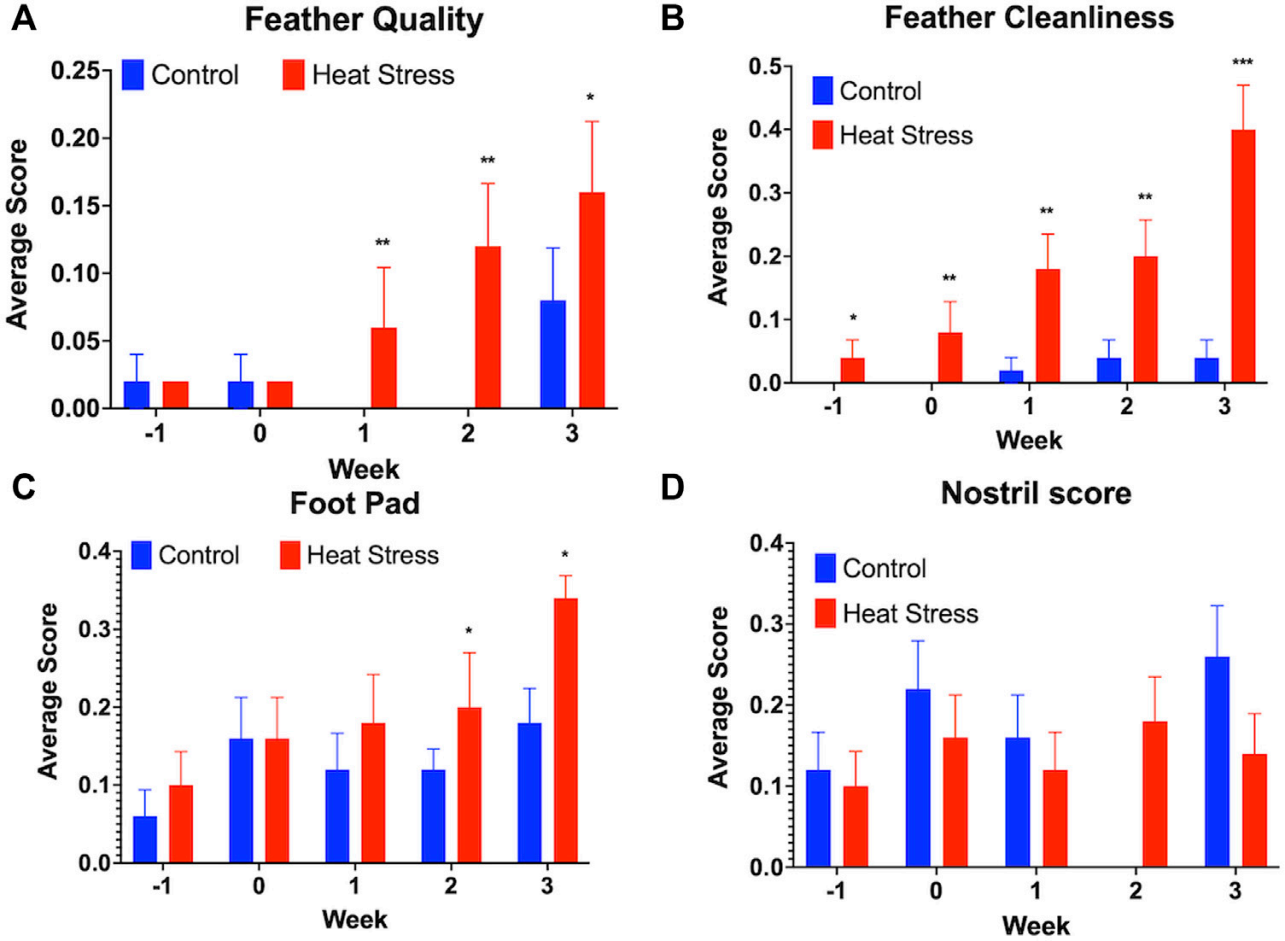
Welfare assessment score for Feather quality **(A)**, Feather cleanliness **(B)**, Foot pad quality **(C)**, and Nostril quality scores **(D)** in ducks exposed to cyclic heat stress (35°C for 10h/day and 29.5°C for the remaining 14h/day) or control. Data shown are means ± SEM, *n* = 65–80/treatment/week. Ducks exposed to heat stress showed higher feather quality scores at all time-point, higher feather cleanliness scores compared to the controls at weeks 1, 2, and 3, and higher foot pad scores compared to the controls at weeks 2, and 3 after onset of treatment and higher scores indicate a decline in welfare. No significant differences in the nostril quality scores were observed between treatment groups. * = *p* < 0.05, ** = *p* < 0.01.

No significant differences were observed in the HLR for either hens (Figure 2A) or drakes (Figure 2B). We observed a significant increase in feather quality scores (*p* < 0.01, Figure 3A), and feather cleanliness scores (*p* < 0.001, Figure 3B), although significant, ducks with feather cleanliness scores below 0.1 are not considered dirty (Fraley et al., 2013; Karcher et al., 2013; Colton et al., 2014). Footpad quality scores (*p* < 0.05, Figure 3C) increased significantly in the HS group compared to controls. Higher BCS indicate a decline in welfare (Fraley et al., 2013; Karcher et al., 2013). All other welfare parameters (Figure 3D) showed no significant differences. Eye score data are not illustrated due to the fact that throughout the experiment, nearly all ducks had “0” scores regardless of treatment, suggesting that the slightly elevated NH_3_ levels in the HS had minimal, or even negligible, confounding effects.

### Egg production, hatchability, and fertility rate

**FIGURE 4:**
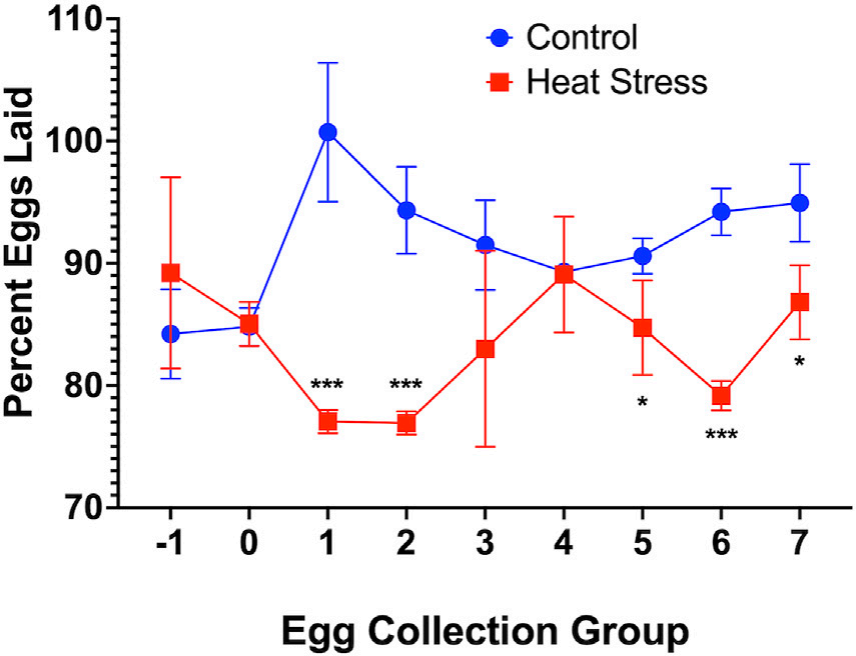
Percent Eggs Laid of Ducks Exposed to Cyclic HS (35°C for 10h/day and 29.5°C for the remaining 14h/day) or control. Data shown are means ± SEM, *n* = 3/time group and all eggs laid over 3 consecutive days averaged per time group. Ducks exposed to heat stress showed a significant lower percent of eggs laid compared to the controls at weeks 1 and 3 after onset of treatment. Egg groups relative to week; groups −1 and 0 (week preceding heat stress), groups 1 and 2 (week 1), groups 3 and 4 (week 2), and groups 5, 6 and 7 (week 3). * = *p* < 0.05, *** = *p* < 0.001.

HS elicited a significant (*p* < 0.001) decrease in egg production at weeks 1 and 3 compared to controls as shown in Figure 4. HS elicited a descriptive decrease in the number of fertile eggs upon candling at 10 days of incubation with values of 98.4% in the control and 92.9% in the HS group, decrease in the number of hatched chicks after the incubation period with values of 76% in the control and 69% in the HS group, and an increase in the number of dead embryo upon hatching with values of 23.2% in the control and 25.7% in the HS group.

### Egg quality

We observed a significant (*p* < 0.001) decrease in shell weight at week 1 only compared to the control. Yolk weight decreased in the HS group but only significant (*p* < 0.05) at week 3 compared to controls. There were no significant differences in other egg quality parameters such as egg weight, shell thickness, shell and vitelline membrane compression strengths. Table 2 illustrates these results.

**TABLE 2:** Effect^a^ of a 3-week chronic cyclic heat stress^b^ on egg quality parameters^c^ of breeder ducks.

	Weeks relative to treatment			
Parameter and treatment group	0	1	2	3
**Egg Weight (g)**				
Control	83.8 ± 0.78	85.8 ± 0.78	86.1 ± 1.00	85.9 ± 1.75
Heat stress	83.8 ± 0.73	83.9 ± 0.96	83.7 ± 0.85	84.4 ± 0.91
**Shell Weight (g)**				
Control	7.96 ± 0.088	8.08 ± 0.072^a^	8.06 ± 0.087	8.26 ± 0.083
Heat stress	8.06 ± 0.101	7.65 ± 0.101^b^	7.80 ± 0.101	8.01 ± 0.095
**Shell Thickness (mm)**				
Control	0.45 ± 0.003	0.45 ± 0.003	0.44 ± 0.003	0.43 ± 0.004
Heat stress	0.45 ± 0.004	0.43 ± 0.004	0.43 ± 0.004	0.44 ± 0.004
**Yolk Weight (g)**				
Control	24.0 ± 0.336	24.2 ± 0.32	24.6 ± 0.30	25.4 ± 0.42^a^
Heat stress	24.0 ± 0.29	24.1 ± 0.39	24.0 ± 0.31	23.6 ± 0.34^b^
**Haugh Unit**				
Control	98.7 ± 0.50	97.1 ± 0.65	95.8 ± 0.95	94.1 ± 0.87
Heat stress	97.8 ± 0.80	99.5 ± 0.69	94.4 ± 0.95	94.1 ± 0.80
**Shell Strength (N)**				
Control	51.3 ± 1.25	50.9 ± 0.99	49.9 ± 1.38	51.9 ± 1.05
Heat stress	47.3 ± 1.81	46.2 ± 1.54	49.0 ± 1.69	48.3 ± 2.11
**Vitelline membrane strength (N)**				
Control	2.04 ± 0.100	2.14 ± 0.136	2.09 ± 0.097	1.89 ± 0.109
Heat stress	1.84 ± 0.115	1.89 ± 0.125	2.23 ± 0.130	1.84 ± 0.106

^a^ Data shown are means ± SEM, *n* = 33–44/treatment/week.

^b^ Breeder ducks exposed to cyclic heat stress (35°C for 10h/day and 29.5°C for the remaining 14h/day) or control measured weekly including week preceding HS.

^c^ The table shows Tukey-kramer pairwise comparisons of HS and control groups. Different letter coding within parameter is significantly different (*p* ≤ 0.05).

### Morphometrics

Hens in the HS group showed a significant decrease in body weight (*p* < 0.001) and number of maturing ovarian follicles (*p* < 0.05) compared to controls. There were no significant differences in the relative weights of spleen or testes in drakes between treatment groups. There were also no significant differences in the relative liver weights of either hens or drakes between treatment groups. Table 3 illustrates these results.

**TABLE 3:** Effect^a^ of a 3-week chronic cyclic heat stress^b^ on immune organ parameters in breeder ducks.

	Week 0		Week 3	
Parameters^c^ ^,^ ^d^	Control	Heat stress	Control	Heat stress
**Hen**				
Body weight (kg)	3.55 ± 0.078	3.38 ± 0.038	3.58 ± 0.057^a^	3.16 ± 0.038^b^
Spleen (g/kg)	0.41 ± 0.028	0.40 ± 0.025	0.48 ± 0.034	0.44 ± 0.018
Follicles (#)	6.1 ± 0.31	5.7 ± 0.45	5.2 ± 0.20	4.4 ± 0.31
Liver (g/kg)	24.3 ± 0.93	24.4 ± 2.03	22.2 ± 0.67	22.6 ± 0.10
**Drake**				
Body weight (kg)	3.71 ± 0.100	3.78 ± 0.160	3.97 ± 0.150	3.56 ± 0.066
Spleen (g/kg)	0.57 ± 0.051	0.54 ± 0.040	0.52 ± 0.017	0.55 ± 0.021
Testes (g/kg)	30.0 ± 3.29	24.2 ± 2.72	25.8 ± 2.58	25.4 ± 1.58
Liver (g/kg)	18.4 ± 0.73	16.5 ± 1.16	15.4 ± 0.77	14.3 ± 0.85

^a^ Data shown are means ± SEM, *n* = 10/treatment for hens and 5/treatment for drakes.

^b^ Breeder ducks exposed to cyclic heat stress (350C for 10h/day and 29.50C for the remaining 14h/day) or control measured 1 week before HS and 3 weeks after.

^c^ The table shows Tukey-kramer pairwise comparisons of HS and control groups. Different letter coding within parameter is significantly different (*p* ≤ 0.05).

^d^ Values of spleen, liver and testes are relative to the body weight (g/kg).

### Mass spectrometry for yolk and albumen GCs

**FIGURE 5:**
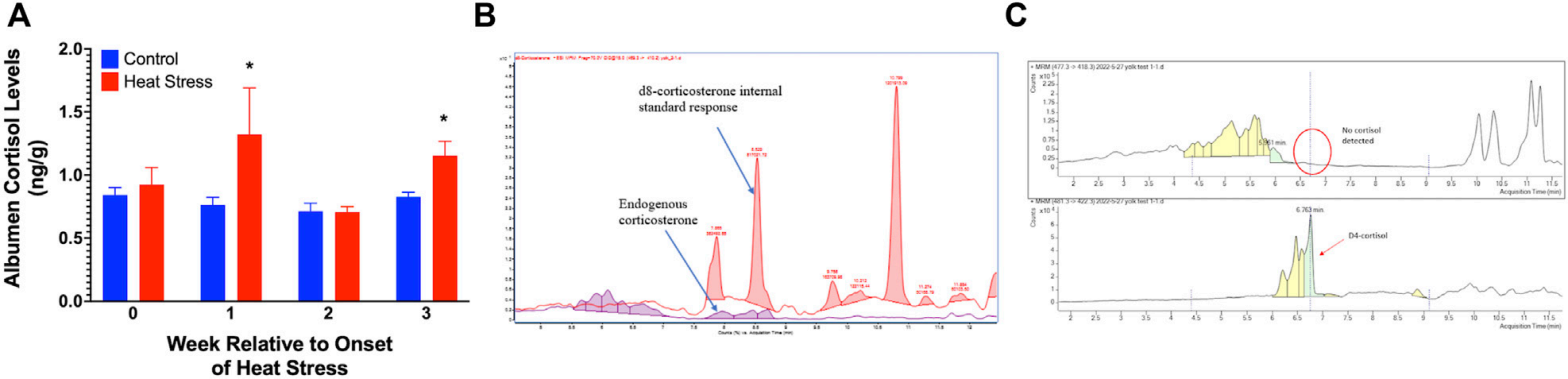
Egg albumen concentration of cortisol **(A)**, Chromatograph overlay for yolk corticosterone comparison **(B)**, and yolk cortisol comparison **(C)** of ducks exposed to cyclic heat stress (35°C for 10h/day and 29.5°C for the remaining 14h/day) or control. Data shown for Figure 5A are means ± SEM, *n* = 6/treatment/week. Albumen cortisol levels were significantly increased at weeks 1 and 3 after onset of treatment compared to controls. The endogenous corticosterone in the samples were below detectable limits. Cortisol and corticosterone were not detected in yolk samples. * = *p* < 0.05.

No measurable levels of corticosterone were observed in the albumen from either control or HS groups. We found a significant increase in the albumen cortisol levels at week 1 (*p* < 0.05) and week 3 (*p* < 0.05, Figure 5A) in the HS group compared to the control. There were no measurable levels of corticosterone (Figure 5B) or cortisol (Figure 5C) in the egg yolk from either treatment group.

## Discussion

The purpose of our study was to determine the effects of HS on reproduction, welfare, and glucocorticoid secretion in the Pekin duck. To achieve this, we treated 160 adult drakes and hens at peak lay to HS or control conditions. We observed a decrease in reproductive parameters in hens as seen by reduced egg production, egg quality variables, and follicle numbers. The decrease in fertilized eggs suggests a decrease in male fertility, however the lack of significant differences in testes size suggest a physiological mechanism not evident in this study. Further a sex difference in GC responses, and cortisol, not corticosterone, is deposited into the egg albumen but not yolk. Our data suggest that the increase in albumen cortisol could elicit epigenetic effects on future generations. Further, we observed sex differences in how HS can affect ducks and that these sex differences may be related to sex differences in the glucocorticoid responses as shown by previous studies from our lab (Tetel et al., 2022a; Tetel et al., 2022b).

Numerous studies have reported that differences in the level of GC in mammalian females are higher than in males (Turner, 1990; Ferrini et al., 1997; Bourke et al., 2012). Madison et al. (2018) demonstrated sex differences in hippocampal responses to stressors in zebra finches. These authors further stated that females showed upregulated hippocampal mineralocorticoid receptors while males downregulated both mineralocorticoid and glucocorticoid receptors in response to social stressors. These findings confirms our results that further suggest a sex difference in the HS response, although, we could have taken earlier blood samples to see the acute phase of GC secretion after the onset of HS. Our lab has consistently observed a sex difference in the time of peak of GC secretion in ducks and we have shown that the peak secretion is very dependent upon when that individual first perceives the onset of stressor, so knowing exactly when to collect early blood samples in this current study to observe peak secretion of GC would have been problematic (Tetel et al., 2022a; Tetel et al., 2022b). The changes in circulating glucocorticoid levels associated with HS is likely related to changes in body morphometrics associated with this stressor.

HS has been shown to cause decrease in the body and organ weights of poultry, as further demonstrated in our study. The decline in body weight can partly be attributed to the reduction in feed intake and impairment of the intestinal integrity (Quinteiro-Filho et al., 2010; Quinteiro-Filho et al., 2012; Sohail et al., 2012; Khatlab et al., 2018). It has been reported that HS negatively affects intestinal mucosa and microbiota composition (Liu et al., 2009; Kers et al., 2018). Further, the damage to mucosal epithelium can directly affect intestinal barrier function, nutrient absorption and impair production performance (Moeser et al., 2007). This finding confirms our result that showed a decrease in the body weight of hens in the HS group. Feed intake data would have added strength to this observation but was, unfortunately, not possible during this study. We also observed a decrease in the number of maturing ovarian follicles, similar to that described by others. Rozenboim et al. (2007) reported the reduction in ovary weight and number of maturing follicles after 6 days of exposure to HS and decline in plasma progesterone and estradiol levels. Other studies also showed a decrease in ovary weight, oviduct weight, hierarchical follicle number and weight following exposure of Japanese quail to HS (Pu et al., 2019). The decline in the number of maturing follicles might be attributed to reduction in blood flow to the ovary during heat stress (Wolfenson et al., 1981). The reduction of maturating follicles in heat stressed hens may also, in part, be caused by the increase in the level of prolactin that decreases the secretion of gonadotropins by suppressing pituitary gonadotrophs leading to ovarian regression (Rozenboim et al., 1993; You et al., 1995).

Several studies have reported the adverse effects of heat stress on egg production and the egg quality of laying hens including a reduction in egg weight, shell strength and weight, albumen deposition, yolk weight, and Haugh unit (Mashaly et al., 2004; Ebeid et al., 2012; Ma et al., 2014; El-Tarabany, 2016; Barrett et al., 2019). These effects are mainly observed in heat-stressed ducks where the decrease in egg quality was attributed to the reduction in feed intake, respiratory alkalosis, and reduction in blood flow to the oviduct (Ma et al., 2014). The decrease in yolk weight was attributed to impairment in the ovary that is caused by the reduction of blood flow to this organ (Mashaly et al., 2004; El-Tarabany, 2016). Ebeid et al. (2012) demonstrated that heat stress leads to respiratory alkalosis due to hyperventilation and the increased blood pH reduces the amount of Ca^+^ that is essential for shell formation. These authors further suggested that the defect in transport of intestinal calbindin leads to a further decrease in calcium absorption. However, others have shown no effects of GC on egg or albumen weight (Wolfenson et al., 1979; Deng et al., 2012). Similar to these previous studies, we found that heat stress decreased shell weight and yolk weight. We conclude that HS affected shell weight and yolk weight but that to achieve decrease in egg weight probably required an extended or greater heat stress exposure in laying ducks, or possibly ducks are less sensitive to HS compared to other poultry species.

Historically, it was suggested that corticosterone is the main plasma GC in birds produced by adrenal cortical cells (Deroos, 1961). A quest to develop a non-invasive measure of stress in chickens lead to the measure of GC in egg albumen and it was discovered that subcutaneous injection of corticosterone leads to its direct deposition in egg (Downing and Bryden, 2008). Campbell et al. (2018) reported an increase in corticosterone deposition in the egg of birds in non-enriched cages compared to birds housed in an enriched environment. However, recently Caulfield and Padula. (2020) showed that it is cortisol that is deposited in albumen, not corticosterone, as suggested by previous studies. Lechner et al. (2001) investigated the presence of steroidogenic enzymes in the Bursa of Fabricius and thymus and reported that the steroidogenic pathways lead to the synthesis of cortisol, and not corticosterone, within these organs. These authors further stated that cortisol has a higher affinity to the GC receptor than does corticosterone in the bursa and thymus of chickens. Our results support the idea that cortisol is a physiologically important part of the stress response in ducks and that cortisol is selectively deposited in egg albumen and could be used as a non-invasive marker of welfare.

To determine if corticosterone is present in egg yolk, we analyzed yolk extract using mass spectrometry and detected extremely low to non-measurable levels of corticosterone and non-detectable levels of cortisol. We conclude that cortisol is not deposited in yolk but might be synthesized and deposited into the albumen during egg formation in the magnum of the oviduct. Therefore, there is need for further research on extra-adrenal synthesis of steroid hormones in the reproductive tract and other tissues. Several studies have reported increase in yolk immunoreactive corticosterone following the administration of corticosterone or exposure to heat, restraint, or rearing system stress (Okuliarová et al., 2010; Hayward et al., 2015; Della Costa et al., 2019; Pu et al., 2019; Miltiadous and Buchanan, 2021). Corticosterone in avian blood is predominantly bound to proteins, such as corticosterone binding globulin (CBG) and albumin which might also be the case for yolk corticosterone (reviewed by Scanes, 2016). Rettenbacher et al. (2005) were unable to demonstrate the presence of corticosterone in the yolk following the administration of radiolabeled corticosterone or injection of ACTH but suggested the presence of gestagens, a class of steroid hormones also known as progestogens, cross-react with corticosterone antibodies and thus responsible for confounding the corticosterone concentration levels. To investigate this possibility, Rettenbacher et al. (2009) analyzed yolk extracts and reported the absence of immunoreactive compound for cortisol, but rather detected concentrations of progesterone, pregnenolone, gestagen metabolites and a very small peak for corticosterone, all of which support the findings in our study. Interestingly, Rettenbacher et al. (2009) further stated that progesterone antibody revealed 3 peaks representing at least 3 immunoreactive steroids while corticosterone antibody detected immunoreactive compounds at the same elution position as progesterone; confirming that gestagens cross-react with corticosterone antibodies. Quillfeldt et al. (2011) also reported low corticosterone and high gestagen concentrations in the yolk of rockhopper penguins and imperial shag egg yolks. Given the high concentration of gestagens compared to other steroid hormones in the yolk (Von Engelhardt and Groothuis, 2005; Quillfeldt et al., 2011) even low cross-reactivity is capable of confounding results, so it is likely that GC activity measured in the yolk by other studies using antibody-based detection systems are due to cross-reactivity with gestagens or their metabolites. There is need for further studies to investigate the cross-reactivity of GC with other steroid hormones and their metabolites in the yolk, and the validation of these antibody-based, steroid hormone detection assays. However, the presence of cortisol in albumen has been confirmed in our lab and by others (Caulfied and Padula, 2020) using mass spectrometry.

The epigenetic effect of maternal glucocorticoids is under current investigation by our lab, as maternal exposure to stressors can alter the phenotype of offspring thereby affecting both pre-hatch and post hatch welfare and development. Several studies have reported the effect of *in ovo* administration of corticosterone on embryonic and post-hatch development of the offspring (Saino et al., 2005; Janczak et al., 2007; Peixoto et al., 2020). In addition, embryonic exposure to corticosterone led to reduced hatchability, body weight, competitive ability, growth rate, increase embryo mortality and fearfulness (Janczak et al., 2006; Eriksen et al., 2010). Hayward and Wingfield. (2004), reported decrease in hatching weight, growth rate, and increase in HPA axis response to restraint stress in the offspring of Japanese quail implanted with corticosterone. Further studies are underway in our lab to explore the effect of maternal GC on F_1_ development, welfare, and adaptation to stress.

In summary, we showed that there are sex differences in GC response of ducks exposed to chronic HS with hens been more responsive to the stressor as evidenced by higher circulating levels of GCs. In addition, we demonstrated the selective deposition of cortisol in egg albumen but not yolk following HS. Therefore, we emphasize the importance of studying the effect of cortisol on embryonic and post-hatch development. Further, HS causes significant decrease in reproductive parameters in hens as seen by reduced egg production, egg yolk, shell weight, and follicle numbers and the decrease in fertility as evident by the reduction in number of fertile eggs and hatchability. Our results suggest that GC elicit differential effects and although corticosterone has been stated to be the predominant GC in avian species, cortisol may provide critical information to further understand and to improve welfare. Finally, the measure of cortisol in egg albumen can be used as a non-invasive marker of stress.

## Data Availability

The raw data supporting the conclusions of this article will be made available by the authors, without undue reservation.
